# Insights into vaccine hesitancy from systems thinking, Rwanda

**DOI:** 10.2471/BLT.20.285258

**Published:** 2021-09-28

**Authors:** Catherine Decouttere, Stany Banzimana, Pål Davidsen, Carla Van Riet, Corinne Vandermeulen, Elizabeth Mason, Mohammad S Jalali, Nico Vandaele

**Affiliations:** a Research Center for Access-to-Medicines, Naamsestraat 69, 3000 Leuven, Belgium.; b East African Community Regional Centre of Excellence for Vaccines, Immunization and Health Supply Chain Management, University of Rwanda, Kigali, Rwanda.; c System Dynamics Group, University of Bergen, Bergen, Norway.; d Leuven University Vaccinology Center, KU Leuven, Leuven, Belgium.; e Massachusetts General Hospital Institute for Technology Assessment, Harvard Medical School, Cambridge, United States of America.

## Abstract

**Objective:**

To investigate vaccine hesitancy leading to underimmunization and a measles outbreak in Rwanda and to develop a conceptual, community-level model of behavioural factors.

**Methods:**

Local immunization systems in two Rwandan communities (one recently experienced a measles outbreak) were explored using systems thinking, human-centred design and behavioural frameworks. Data were collected between 2018 and 2020 from: discussions with 11 vaccination service providers (i.e. hospital and health centre staff); interviews with 161 children’s caregivers at health centres; and nine validation interviews with health centre staff. Factors influencing vaccine hesitancy were categorized using the 3Cs framework: confidence, complacency and convenience. A conceptual model of vaccine hesitancy mechanisms with feedback loops was developed.

**Findings:**

A comparison of service providers’ and caregivers’ perspectives in both rural and peri-urban settings showed that similar factors strengthened vaccine uptake: (i) high trust in vaccines and service providers based on personal relationships with health centre staff; (ii) the connecting role of community health workers; and (iii) a strong sense of community. Factors identified as increasing vaccine hesitancy (e.g. service accessibility and inadequate follow-up) differed between service providers and caregivers and between settings. The conceptual model could be used to explain drivers of the recent measles outbreak and to guide interventions designed to increase vaccine uptake.

**Conclusion:**

The application of behavioural frameworks and systems thinking revealed vaccine hesitancy mechanisms in Rwandan communities that demonstrate the interrelationship between immunization services and caregivers’ vaccination behaviour. Confidence-building social structures and context-dependent challenges that affect vaccine uptake were also identified.

## Résumé

**Objectif:**

Analyser l'hésitation vaccinale menant à une immunisation insuffisante et à une épidémie de rougeole au Rwanda, mais aussi développer un modèle conceptuel de facteurs comportementaux à l'échelle communautaire.

**Méthodes:**

Nous avons examiné les systèmes d'immunisation locaux dans deux communautés rwandaises (l'une ayant récemment subi une épidémie de rougeole) en utilisant la pensée systémique, une approche centrée sur l'humain et des cadres comportementaux. Les données ont été récoltées entre 2018 et 2020 auprès de plusieurs sources: les discussions avec 11 prestataires de services liés à la vaccination (hôpitaux et centres médicaux); des entretiens avec 161 aides-soignants du département pédiatrique dans divers centres médicaux; et enfin, neuf entretiens de validation avec le personnel des centres médicaux. Nous avons classé les facteurs conditionnant l'hésitation vaccinale conformément à la règle des 3 C: confiance (*confidence*), sous-estimation du danger (*complacency*) et commodité (*convenience*). Un modèle conceptuel des mécanismes d'hésitation vaccinale assorti de boucles de rétroaction a également été élaboré.

**Résultats:**

D'après une comparaison entre les perspectives des prestataires de services et aides-soignants, tant en milieu rural que périurbain, des facteurs similaires renforcent la prise vaccinale: (i) un degré de confiance élevé envers les vaccins et les prestataires de services, fondé sur les relations entretenues avec le personnel des centres médicaux; (ii) le rôle d'intermédiaire joué par les agents de santé communautaires; et enfin, (iii) un fort sentiment d'appartenance à la collectivité. Les facteurs qui ont tendance à accroître l'hésitation vaccinale (parmi lesquels figurent l'accessibilité aux services et l'absence de suivi adéquat) varient entre prestataires de services et aides-soignants, ainsi que selon les milieux. Le modèle conceptuel pourrait servir à identifier les moteurs de la récente épidémie de rougeole, et orienter les actions destinées à améliorer la prise vaccinale.

**Conclusion:**

L'application des cadres comportementaux et de la pensée systémique ont révélé les mécanismes qui sous-tendent l'hésitation vaccinale au sein des communautés rwandaises, et qui prouvent la corrélation entre les services d'immunisation et le comportement des aides-soignants en la matière. Nous avons également découvert des structures sociales de restauration de la confiance ainsi que des défis qui, en fonction du contexte, ont un impact sur la prise vaccinale.

## Resumen

**Objetivo:**

Estudiar la reticencia a las vacunas que ha causado la falta de vacunación y la aparición de un brote de sarampión en Ruanda, y desarrollar un modelo conceptual a nivel comunitario de los factores de comportamiento.

**Métodos:**

Se analizaron los programas locales de vacunación en dos comunidades de Ruanda (una de las que experimentó un brote de sarampión hace poco) mediante el uso de marcos de pensamiento sistémico, de diseño centrado en el ser humano y de comportamiento. Los datos se recopilaron entre 2018 y 2020 a partir de conversaciones con 11 proveedores de servicios de vacunación (es decir, personal de hospitales y de centros sanitarios); entrevistas con 161 cuidadores de niños en centros sanitarios; y 9 entrevistas de validación con personal de centros sanitarios. Los factores que influyen en la reticencia a la vacunación se clasificaron mediante el modelo de las 3 C: confianza, complacencia y conveniencia. Asimismo, se elaboró un modelo conceptual de los mecanismos de reticencia a la vacunación que incluye ciclos de retroalimentación.

**Resultados:**

Una comparación entre las perspectivas de los proveedores de servicios y de los cuidadores, tanto en entornos rurales como periurbanos, indicó que factores similares reforzaban la adopción de las vacunas: i) la gran confianza en las vacunas y en los proveedores de servicios gracias a las relaciones personales con el personal del centro sanitario; ii) el rol de conexión de los profesionales sanitarios de la comunidad; y iii) el gran sentido de pertenencia a la comunidad. Los factores identificados que aumentan la reticencia a las vacunas (por ejemplo, la accesibilidad a los servicios y el seguimiento insuficiente) difieren entre los proveedores de servicios y los cuidadores y entre los distintos entornos. El modelo conceptual se podría utilizar para explicar los factores que influyeron en el reciente brote de sarampión y para orientar las intervenciones diseñadas con el objetivo de aumentar la adopción de vacunas.

**Conclusión:**

La aplicación de marcos de comportamiento y del pensamiento sistémico reveló mecanismos de reticencia a las vacunas en las comunidades de Ruanda que evidencian la interrelación entre los servicios de vacunación y el comportamiento de vacunación de los cuidadores. También se identificaron las estructuras sociales de fomento de la confianza y los desafíos específicos del contexto que afectan a la adopción de las vacunas.

## ملخص

**الغرض:**

الاستقصاء عن التردد في تعاطي اللقاح وهو ما يؤدي إلى نقص المناعة وتفشي مرض الحصبة في رواندا، وتطوير نموذج مفاهيمي على مستوى المجتمع للعوامل السلوكية.

**الطريقة:**

تم استكشاف أنظمة التحصين المحلية في مجتمعين روانديين (تعرض أحدهما مؤخرًا لتفشي مرض الحصبة)، باستخدام التفكير النظامي، والتصميم المرتكز على الإنسان، وأطر العمل السلوكية. تم تجميع البيانات بين عامي 2018 و2020 من: مناقشات مع 11 من مقدمي خدمة التطعيم (أي فريق العمل بالمستشفى والمركز الصحي)؛ ومقابلات شخصية مع 161 من مقدمي الرعاية للأطفال في المراكز الصحية؛ وتسع مقابلات شخصية للتحقق مع فريق عمل المركز الصحي. تم تصنيف العوامل التي تؤثر على التردد في تعاطي اللقاح باستخدام إطار عمل 3Cs: الثقة (Confidence)، والرضا (Complacency)، والراحة (Convenience). تم تطوير نموذج مفاهيمي لآليات التردد في تعاطي اللقاح مع حلقات طرح التعليقات.

**النتائج:**

إن المقارنة بين وجهات نظر مقدمي الخدمات، ووجهات نظر مقدمي الرعاية، في كل من المناطق الريفية وشبه الحضرية، أظهرت أن هناك عوامل مماثلة قد زادت من نسبة تعاطي اللقاح: (1) ثقة عالية في اللقاحات ومقدمي الخدمة على أساس العلاقات الشخصية مع فريق عمل المركز الصحي؛ و(2) الدور المتصل للعاملين الصحيين بالمجتمع؛ و (3) شعور قوي بالانتماء للمجتمع. إن العوامل التي تم التوصل إلى أنها تزيد من التردد في تعاطي اللقاح (مثل إمكانية الحصول على الخدمة، والمتابعة غير الكافية)، اختلفت بين مقدمي الخدمة ومقدمي الرعاية، وفيما بين المواقع. يمكن استخدام النموذج المفاهيمي لشرح دوافع تفشي مرض الحصبة مؤخرًا، ولتوجيه التدخلات المقررة لزيادة نسبة تعاطي اللقاح.

**الاستنتاج:**

إن كل من تطبيق أطر العمل السلوكية والتفكير النظامي كشفا عن آليات التردد في تعاطي اللقاح في المجتمعات الرواندية، والتي توضح العلاقة المتبادلة بين خدمات التحصين وسلوك التطعيم لدى مقدمي الرعاية. كما تم التوصل إلى الهياكل الاجتماعية لبناء الثقة، والتحديات التي تعتمد على السياق، والتي تؤثر على نسبة تعاطي اللقاح.

## 摘要

**目的:**

调查导致卢旺达免疫不足和麻疹爆发的“疫苗犹豫”，并开发一个社区层面的概念性行为因素模型。

**方法:**

采用系统思维、以人为本的设计理念和行为框架，探索了卢旺达两个社区（其中一个地区最近经历了麻疹爆发）的当地免疫系统。收集的 2018 年至 2020 年数据来自：与 11 位疫苗接种服务提供者（即医院和卫生中心工作人员）的讨论；采访了卫生中心的 161 名儿童看护人员；以及与卫生中心工作人员进行的九次验证访谈。使用 3C 框架对影响“疫苗犹豫”的因素进行分类：信心感、满足度和便利性。开发了具有反馈回路的“疫苗犹豫”机制的概念模型。

**结果:**

对农村和城郊环境中服务提供者和看护人员的观点进行比较，结果表明以下类似因素加强了疫苗接种：(I) 基于与卫生中心工作人员的个人关系对疫苗和服务提供者的高度信任；(ii) 社区卫生工作者所起到的纽带作用；(iii) 强烈的社区意识。被确定为增加“疫苗犹豫”的因素（例如服务的可及性和后续随访不足）在不同的环境中以及在不同的服务提供者和看护人员之间有所差异。该概念模型可用于解释近期麻疹爆发的驱动因素，并指导提高疫苗接种率的干预措施。

**结论:**

行为框架和系统思维的应用揭示了卢旺达社区的“疫苗犹豫”机制，证明了免疫服务与护理人员的疫苗接种行为之间的相互关系。还确定了营造信心的社会结构和不同环境下影响疫苗接种的挑战。

## Резюме

**Цель:**

Изучить недоверие к вакцинации, ведущее к недостаточной иммунизации и вспышке кори в Руанде, а также разработать концептуальную модель поведенческих факторов на общинном уровне.

**Методы:**

Были изучены местные системы по иммунизации в двух руандийских общинах (одна из которых недавно пережила вспышку кори) с использованием системного мышления, дизайна, ориентированного на потребности человека, и поведенческих структур. Были собраны данные в период с 2018 по 2020 год в результате обсуждений с 11 поставщиками услуг в области вакцинации (то есть персоналом больниц и центров здравоохранения), интервью с 161 лицом, осуществляющим уход за детьми в центрах здравоохранения, и девяти оценочных интервью с персоналом центра здравоохранения. Факторы, влияющие на недоверие к вакцинации, были классифицированы с использованием «системы 3 факторов»: доверие, удовлетворенность жизненной ситуацией и удобство. Была разработана концептуальная модель механизмов недоверия к вакцинации с обратной связью.

**Результаты:**

Сравнение мнений поставщиков услуг и лиц, осуществляющих уход, в сельских и пригородных районах показало, что увеличению охвата вакцинацией способствовали аналогичные факторы: (i) высокое доверие к вакцинам и поставщикам услуг, основанное на личных отношениях с персоналом центра здравоохранения; (ii) связующая роль местных медицинских работников; (iii) сильное чувство общности. Факторы, идентифицированные как растущее недоверие к вакцинации (например, доступность обслуживания и недостаточное последующее наблюдение), различались между поставщиками услуг и лицами, осуществляющими уход, а также между районами. Концептуальную модель можно использовать для объяснения причин недавней вспышки кори и для разработки мероприятий, направленных на увеличение охвата вакцинацией.

**Вывод:**

Применение поведенческих структур и системного мышления выявило в руандийских общинах механизмы недоверия к вакцинации, которые демонстрируют взаимосвязь между иммунизационным обслуживанием и поведением лиц, осуществляющих уход, в отношении вакцинации. Были также определены социальные структуры, способствующие укреплению доверия, и контекстно зависимые проблемы, влияющие на охват вакцинацией.

## Introduction

Rwanda has a strong immunization system,^1^ a well-organized vaccine supply system and a well-functioning community health worker (CHW) programme.^2^ As a result, national immunization coverage rates are high and new vaccines are introduced swiftly.^3^^,^^4^ Nevertheless, some communities still face local disease outbreaks due to underimmunization. A major driver of underimmunization is vaccine hesitancy,^5^ which is defined by the World Health Organization (WHO) as “delay in acceptance or refusal of vaccination despite availability of vaccination services.”^6^ According to WHO, “vaccine hesitancy is complex and context specific, varying across time, place, and vaccines” and is one of the top 10 global health threats.^6^^,^^7^

Factors influencing vaccine hesitancy can be categorized using the 3Cs framework: (i) confidence; (ii) complacency; and (iii) convenience.^8^ As psychological, sociological and environmental drivers are paramount in instigating the behavioural changes required to address vaccine hesitancy,^9^^,^^10^ understanding the contextual relationships between vaccine hesitancy and socioeconomic determinants of health is crucial.^11^^–^^16^ Additionally, better understanding of vaccine hesitancy is needed to achieve the sustainable development goals, including universal access to quality vaccines.^17^ WHO’s Immunization Agenda 2030 highlights the importance of people-centredness for understanding the context-specific root causes of vaccine hesitancy and for co-designing solutions.^18^^,^^19^ However, the literature has major gaps on: (i) the analysis of human-centred design approaches; (ii) the relationship between beneficiaries and service delivery; and (iii) incorporating the interaction between hesitancy factors into policy and intervention design.^6^

For our study of underimmunization drivers in a low-income country, we focused on measles in Rwanda, because: (i) measles is highly contagious and there were indications of underimmunization; (ii) measles increases morbidity and mortality because it erases the immune memory and increases susceptibility to other infectious diseases;^20^^,^^21^ (iii) despite long-standing vaccination programmes, eradication has not been achieved globally according to the Global Measles and Rubella Strategic Plan 2012–2020;^22^ and (iv) the second measles vaccine dose was designated a performance tracer in the Immunization Agenda 2030.^18^ Moreover, the measles programme is monitored by WHO and the Rwandan government, which can provide data for future quantitative models. In Rwanda, the measles vaccination programme has been quite successful: it achieved a national coverage rate above 85% for the second measles and rubella vaccine dose in 2015 and reached the target national coverage rate of 95% for the first dose in 2017.^18^^,^^23^ The first measles and rubella vaccine dose is administered 9 months after birth and, since 2015, the second dose is administered 15 months after birth. Despite these achievements, Rwanda still faces sporadic measles outbreaks (e.g. in Western Province in 2019).^24^

To inform policy design and to improve immunization levels, we aimed to study the mechanisms underlying vaccine hesitancy in Rwanda that contributed to local underimmunization for measles and a subsequent measles outbreak. We conducted an analysis of immunization service delivery in both a rural community and a peri-urban community between 2018 and 2020 and derived a conceptual model of vaccine hesitancy to assist in the design of sustainable interventions.

## Methods

**Fig. 1:**
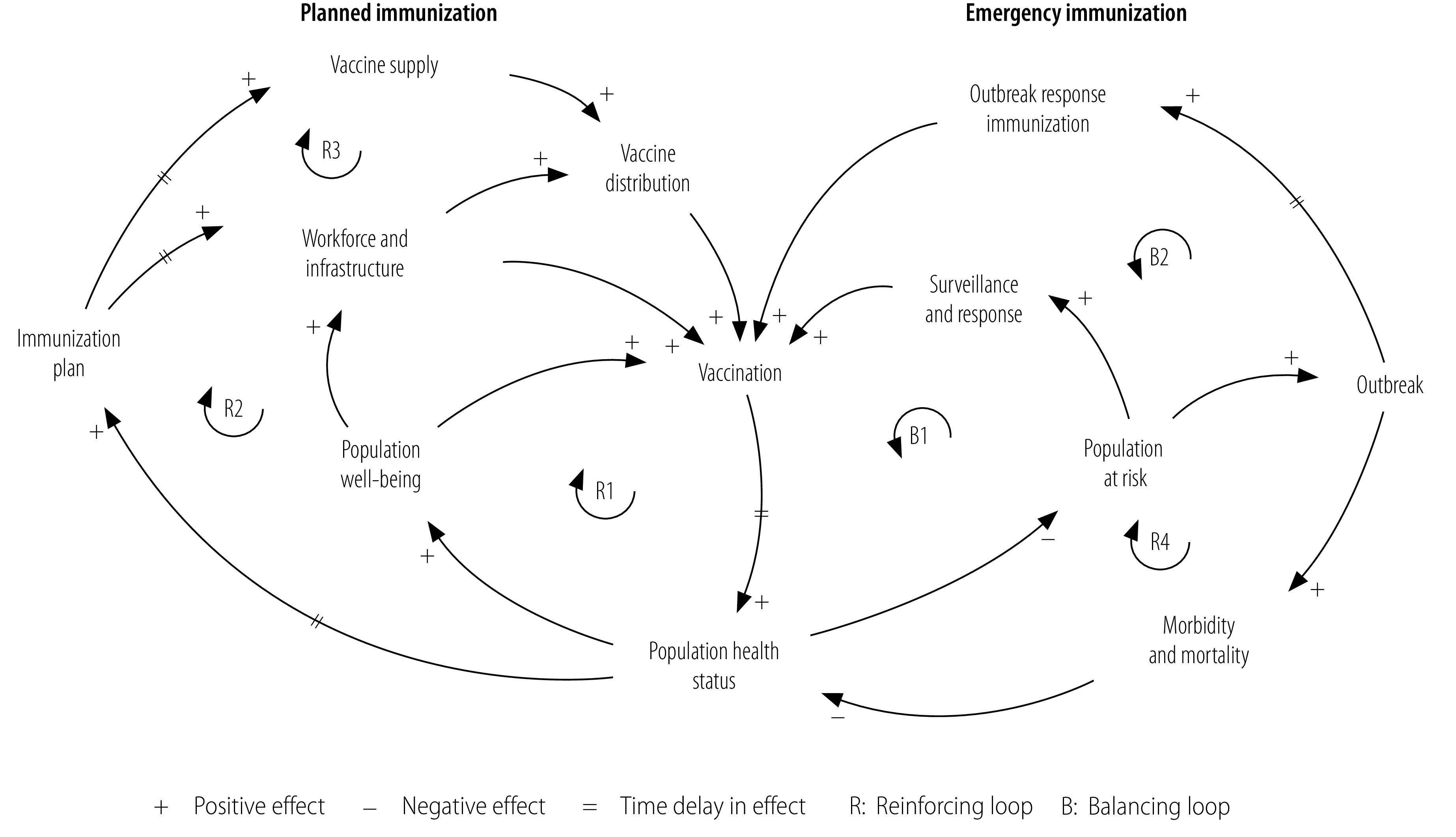
Immunization system diagram

Notes: The immunization system diagram is a high-level conceptual model of a national immunization system that was developed to engage stakeholders in immunization system assessment and sustainable redesign.^25^ The left side illustrates planned immunization (i.e. routine immunization and planned immunization campaigns) guided by a national immunization plan, and the right side illustrates emergency immunization (i.e. immunization in response to disease outbreaks). Loops R1 to R4 are reinforcing feedback loops that cause change in the same direction and loops B1 and B2 are balancing loops that cause change in the opposite direction. See Decouttere et al. for more information.^25^ The focal point of the immunization system diagram is vaccination and all efforts in the system are geared towards service delivery. Vaccination depends on three fundamental so-called flows that need to be synchronized at every vaccination session: (i) child (represented in the diagram by population well-being); (ii) nurse (represented by workforce and infrastructure); and (iii) vaccine (represented by vaccine distribution). Successful vaccination leads to better population health status, although only after a delay. From population health status, there are several loops that affect both planned and emergency immunization, but in different ways.

Our study design was based on WHO’s framework for health system building blocks and an already published immunization system diagram (Fig. 1).^25^^,^^26^ In analysing vaccine uptake, we applied systems thinking and behavioural frameworks such as the 3Cs framework and the behavioural drivers theory.^8^^,^^9^^,^^26^^–^^31^ First, we assessed how underimmunization was influenced by the three so-called immunization service flows: (i) the vaccinee (child); (ii) the health-care workforce (nurse); and (iii) vaccine availability (vaccine). Second, we conducted in-depth interviews with health centre staff and collected secondary data (e.g. on vaccine orders, inventories and disease outbreaks). Based on our findings, we interviewed children’s caregivers to understand local factors influencing vaccine hesitancy. Then, we used the 3Cs framework to categorize factors reported by caregivers and health centre staff.^6^ Finally, we derived causal relationships between behavioural drivers, vaccination intent and vaccination uptake by analysing the vaccine hesitancy factors identified and present these relationships in causal loop diagrams. These diagrams are helpful for understanding complex systems and for developing interventions.^29^^,^^30^ All causal relationships in the causal loop diagrams and vaccine hesitancy factors were validated and explained during additional discussions with health centre staff.

### Participants

We interviewed 11 vaccinators and staff of the Expanded Programme on Immunization at three district hospitals, six health centres in a peri-urban setting in Kicukiro District, Kigali Province and five health centres in a rural setting in Ngororero District, Western Province. Additional details are available from the data repository.^32^ At the community level, we interviewed caregivers at two health centres: one in rural Ramba in Ngororero District, where there was a measles outbreak in 2019, and one in Gahanga in the Kicukiro District. After a measles outbreak in July 2019, one of the Ramba health centre’s outreach posts became the Sovu health centre to be closer to people in the catchment area. Although these two locations share the same climate, epidemiological characteristics and health systems, they have different geographical and socioeconomic characteristics (Table 1). We attended two vaccination sessions at health centres and one outreach vaccination session in both communities. Each caregiver present was invited to participate in the study (i.e. a convenience sample) by having a face-to-face interview in or near the session waiting room. After the purpose of the research was explained, 161 caregivers participated: they were predominantly female and included both experienced mothers and mothers of firstborns.

**Table 1:** Characteristics of two participating communities, study of vaccine hesitancy, Rwanda, 2018–2020

Characteristic	Community	
	Ramba and Sovu^a^	Gahanga
Geographical context	Rural community, hilly and remote landscape, limited road infrastructure and long distances to the health centre (i.e. more than 5 km or 2 hours travel time)	Peri-urban community with relatively short distances to the health centre (i.e. less than 5 km or 2 hours travel time)
Socioeconomic context	22% of the population live in extreme poverty,^33^ and many men are employed in coltan mining	9% of the population live in extreme poverty,^33^ and the affordability of transport is less problematic than in Ramba or Sovu
Location of local district hospital	Kabaya, Ngororero District, Western Province	Masaka, Kicukiro District, city of Kigali
Population	64 000 (50% Ramba health centre and 50% Sovu health centre)	67 000
Weekly vaccination sessions at fixed locations or outreach posts	2–4 at the Ramba health centre and 1–3 at the Sovu health centre (since 1 January 2020)	1–2 (fewer outreach services due to shorter distances and more affordable transport)
Measles outbreak since 2018	July 2019 in the Sovu catchment area	None
Migration	No significant in or out migration	Increasing number of people settling in the area as they flee Kigali’s city centre where property prices are continuously increasing. Some clients visited health centres other than the one they were assigned to

^a^ After a measles outbreak in July 2019 at one of the Ramba health centre’s outreach posts, that post became the Sovu health centre to be closer to people in the catchment area.

### Data collection and analysis

In-depth discussions were conducted with staff at the 11 health centres (data repository)^32^ between September 2018 and November 2019 by three senior researchers and between December 2019 and February 2020 by one researcher. Discussions lasted 60 to 120 minutes and followed a topic guide (more details available in the data repository).^32^ Four researchers analysed data in field notes and photographed documents.

After one day’s training, six Rwandan data collectors conducted semistructured interviews with caregivers in November 2020 (data repository).^32^ Conversations lasting 7 to 12 minutes were held in *Kinyarwanda*, translated into English by the interviewer and later discussed with a supervisor and the research team to reach a consensus on interpretation. Then, two researchers analysed factors associated with vaccine hesitancy using Excel (Microsoft Corporation, Redmond, United States of America) and four researchers classified these factors. One researcher held additional discussions with health centre staff between April and December 2020 to validate the findings (data repository).^32^

Three researchers used the main underlying mechanisms of vaccine hesitancy identified by the analysis to construct causal loop diagrams, which illustrate the interconnections between different factors and show feedback loops. The feedback loops can be either reinforcing (i.e. cause change in the same direction) or balancing (i.e. cause change in the opposite direction). The study was approved by the Rwanda National Ethics Committee (No. 195/RNEC/2019) and is reported according to consolidated criteria for reporting qualitative research.^34^

## Results

During data collection at the 11 health centres, we found no evidence of vaccine stock-outs in 2018 or 2019. Moreover, there were no human capacity limitations that resulted in immunization services being unavailable and national immunization coverage rates were above 90%. However, measles outbreaks occurred because of a lack of timely immunization. The suspected cause was reluctant vaccine uptake rather than limited vaccine availability.

### Vaccine hesitancy

#### Service providers’ perspective

Interviews with Expanded Programme on Immunization staff at the Ramba and Sovu health centres, the Gahanga health centre and nine neighbouring health centres revealed contextual and behavioural factors that contributed to vaccine hesitancy. These factors were categorized according to the 3Cs framework (i.e. confidence, complacency and convenience) using a determinants matrix (Table 2; available at https://www.who.int/publications/journals/bulletin/).^6^ Vaccination service providers reported that confidence is increased by trust in immunization service delivery and that a good relationship between caregivers and both nurses and CHWs is key. However, the time health centre staff could spend with each caregiver and the resulting quality of care were affected by an increased workload due to high peri-urban immigration rates of up to 10% per year, paperwork and other responsibilities.

**Table 2:** Vaccination service providers’ comments on factors affecting vaccine hesitancy, Rwanda, 2018–2020

Factor affecting vaccine hesitancy^a^	Selected comments on factor by service providers^b^		
	Location of service providers’ facilities^c,d^		
	District hospitals (*n* = 3)	Rural health centres (*n* = 5)	Peri-urban health centres (*n* = 6)
**Confidence**			
Trust in the effectiveness and safety of vaccines and in their manufacturers	Positive: “vaccine hesitancy, in the narrowest sense of trust in the vaccine, is not an issue in Rwanda”	Positive: (i) high level of trust in vaccination; and (ii) one vaccinator saw very few adolescent girls who feared the HPV vaccine and had questions about rumours that the vaccine would prevent pregnancy	Positive: high level of trust in vaccination
Trust in, and personal experience of, the health system and health professionals	Neutral: CHWs are highly respected (more in rural than urban areas). Negative: (i) high turnover of staff at health centres; (ii) insufficient training of vaccinators to upgrade their knowledge; (iii) insufficient induction for newly recruited nurses and vaccinators; (iv) shortage of vaccinators and nurses; and (v) high vaccinator workload due to monitoring and paperwork	Neutral: some caregivers travel far to see an experienced nurse they know rather than attend a new, closer health centre. Negative: (i) shortage of staff at health centres because nurses prefer to get jobs at a hospital or in cities rather than in rural areas; (ii) insufficient training of nurses in vaccine management; and (iii) during lockdown, people were afraid of getting infected with SARS-CoV-2 when they visited health centres	Positive: connections with health centres made during antenatal care and institutional births. Neutral: the impact of lockdown on health centre visits was higher in rural areas. Negative: (i) insufficient time with caregivers because of paperwork load; (ii) too much work for the staff available; and (iii) staff shortages a huge problem
**Complacency**			
Communication and media environment	Positive: mother-and-child health weeks held every 6 months	Positive: CHWs’ role increased during lockdown (when they were the only channel for information)	None
Influential leaders, immunization programme gatekeepers and vaccination lobbies	Positive: health minister supportive of immunization programme, CHWs and mother-and-child health weeks	None	None
Religion, culture, gender and socioeconomic factors	None	Positive: *umuganda* (i.e. monthly Rwandan voluntary community meetings where local issues and updates are discussed). Negative: (i) *umuganda* was temporarily suspended during lockdown; (ii) “mothers are forgetting about the second measles dose”; (iii) “attention of mother reduces when child grows up”; (iii) mothers have other priorities and postpone visits to health centres and vaccination sessions, resulting in late immunization; (iv) “forgetting is not about being unwilling, but the mother couldn’t go because of other priorities”; (v) “I would go tomorrow or next week”; (vi) sometimes the vaccination session is the same day when salaries for 15 days are given out, so caregivers cannot attend that day; and (vii) poor families work long hours away from home, leaving little time for children	None
Knowledge and awareness	Positive: (i) CHWs have an important role in building community engagement; (ii) CHWs are trusted and highly respected by the community; and (iii) home visits by CHWs at childbirth connects mothers with health centres and informs them about immunization. Negative: CHW service may not be sustainable because replacing ageing CHWs depends on the motivation of the next generation	Positive: (i) 45- to 60-minute information sessions before vaccination sessions at health centres and outreach posts; and (ii) very low percentage of people have little understanding of the importance of vaccines. Negative: (i) information sessions at facilities were suspended during lockdown if there was insufficient space; (ii) there was a long interval with no contact with health centres between childhood vaccinations at 9 and 15 months of age; and (iii) during the 2019 measles outbreak, CHWs were not able to visit the whole area to raise awareness and detect measles cases	None
Perceived risks and benefits	None	Negative: (i) the perceived risk of disease was low because cases in the community remained undetected; and (ii) “measles cases were not detected, not diagnosed, and not medically treated”	Positive: “people are intrinsically motivated for vaccination”
Immunization as a social norm	Positive: immunization discussed as part of monthly community meetings (*umuganda*) and mothers’ evenings (*w'ababyeyi*)	None	None
**Convenience**			
Availability of the immunization service	Positive: CHWs’ role in raising awareness in the community and organizing outreach services. Negative: higher vaccine wastage at outreach posts	Negative: (i) poor families were not able to afford travel to health centres and relied on outreach services; (ii) measles and rubella vaccine available only at health centres and not at outreach posts; (iii) BCG vaccine available only when 10 children were waiting; (iv) mothers that gave birth sometimes needed to come back to the health centre within 2 weeks for the BCG vaccine; and (v) vaccines in multidose vials (e.g. the measles and rubella vaccine) were offered once a week whereas other vaccines were offered daily	Positive: (i) outreach services were offered at two sites (2 days per week at one and 1 day per week at the other); (ii) CHWs helped increase the efficiency of outreach services by making sure the right amount of vaccine was taken to outreach posts; and (iii) all vaccines, including the BCG vaccine, offered every day. Negative: vaccines in multidose vials (e.g. the measles and rubella vaccine) were offered only once a week whereas other vaccines were offered daily
Affordability of the immunization service	None	Negative: poor families were not able to afford to travel to health centres	Positive: “poverty is not a reason for not coming, everybody comes: poor and less poor.” Negative: “usually, living conditions are a driver for dropping out”
Geographical accessibility	None	Positive: “short distance, people from different catchment areas and districts come here.” Negative: (i) health centre difficult to access; and (ii) hilly area with long distances to health centres, so people rely on outreach	Negative: restricted access to one health centre due to a landslide
Ability to understand (i.e. language and health literacy)	Negative: mothers sometimes forget the scheduled appointment for the next vaccines	Negative: (i) confusion about the second measles and rubella vaccine dose for children aged 15 months because mosquito nets are dispensed when they are aged 9 months; (ii) mothers’ lack of education is a reason for the second measles and rubella vaccine dose being missed; and (iii) some caregivers are not able to read the vaccination card because of a lack of education	Positive: appointment system with cards is well understood by caregivers. Negative: misunderstanding of practical and organizational policies makes people feel unwelcome
Quality of the service (perceived or real)	None	Positive: (i) CHWs closely involved in outreach organization and in tracing vaccine defaulters; and (ii) fewer patients attended health centres during lockdown, leaving more time for each patient. Negative: (i) high nurse workload due to combining vaccination sessions at health centres and outreach posts with night shifts; (ii) increased number of children to be vaccinated in each session; (iii) one health centre did not function well in the past as it provided only fixed sessions and had no outreach for several years; and (iv) 73 children missed the second measles and rubella vaccine dose in 2018	Positive: “splitting up the large catchment area led to better management. All vaccines are now offered every day, except the BCG vaccine.” Negative: (i) “sometimes the EPI nurse has a night shift the day before the session. Sometimes she cannot attend the session”; (ii) the population is increasing at 10–15% per annum but services cannot keep up; and (iii) experienced mothers saw a drop in service quality due to increased nurse workload as more programmes were decentralized to health centres and health posts (e.g. malaria, tuberculosis and HIV services and family planning)
Convenient time (including waiting time), place and cultural context	Negative: (i) missed opportunities to vaccinate because vaccination days were different in different health centres; and (ii) COVID-19 restrictions resulted in a lack of indoor waiting rooms, making it hard for caregivers and children	Positive: (i) providing nutrition services and family planning in addition to vaccination was received positively; and (ii) having vaccination sessions on market days encourages mothers to attend. Neutral: vaccination is the mother’s responsibility and she prefers to come to the health centre when there is a market nearby. Negative: problem with power backup at a health centre	Positive: providing nutrition services in addition to vaccination. Neutral: the day of the week is less important than whether it is raining. Negative: (i) with 40–50 people per session, waiting times can be up to 5 hours; (ii) 60–75 or even 100 children per session; and (iii) nurse wants to give BCG vaccine and measles and rubella vaccine on the same day but BCG vaccines take time as they require a new file and card to be made each time
Design of vaccination programme, vaccination schedule and data management at health centres	Positive: the transition to a digital data management system will improve the functioning of vaccination sessions	Negative: (i) “defaulter tracing didn’t work well [due to paper-based data system and high workload] and people are lost to follow-up when they go to the neighbouring district”; and (ii) data management system does not show when people are dropping out	Positive: “Computers will be installed soon.” Negative: (i) identifying drop-outs using paper files is labour-intensive and requires the help of CHWs; (ii) “drop-outs are checked at the end of the month, reasons for dropping out are explored. They are usually living conditions, or when people move to another area”; (iii) calculating vaccine coverage is difficult because of the changing population in the catchment area; (iv) at the age of 9 months, children need to get the first measles and rubella vaccine dose at their own health centre to receive a mosquito net; and (v) “no free choice of health centre for measles and rubella”

BCG: bacillus Calmette–Guérin; CHW: community health worker; COVID-19: coronavirus disease 2019; EPI: Expanded Programme on Immunization; HIV: human immunodeficiency virus; HPV: human papillomavirus; SARS-CoV-2: severe acute respiratory syndrome coronavirus 2.

^a^ Factors were categorized using the 3Cs framework as related to confidence, complacency or convenience.^8^

^b^ Comments were classified as positive, neutral or negative with regard to their implications for vaccine uptake.

^c^ Vaccinators and Expanded Programme on Immunization staff were interviewed.

^d^ Details of which interviewees made each comment are available from supplement 3 in the data repository.^32^

We found that complacency was successfully reduced by: (i) organizing 6-monthly mother-and-child health weeks; (ii) regular community meetings (*umuganda* and *w'ababyeyi* or mothers’ evenings); (iii) educational sessions before vaccination sessions; and (iv) individual contacts between CHWs and caregivers. However, in rural settings, mothers tended to deprioritize or forget immunization of older children because of other tasks and because they had little contact with health centres in the 6-month interval between the first and second measles and rubella vaccine doses. This factor increased complacency and, combined with low disease surveillance in Sovu (which led to undetected measles cases), contributed to the 2019 measles outbreak. CHWs were regarded as playing a crucial role in connecting mothers to antenatal care and vaccination services. However, concerns were raised about the sustainability of CHW programmes.

With regard to convenience, the availability of immunization services was negatively impacted by the supply of measles and rubella vaccine being limited outside of health centres due to a desire to reduce wastage of multidose vials. In addition, poor families, particularly in rural areas, were unable to attend sessions at health centres because of travel costs, distance, safety or the time required. In peri-urban settings and at district and central levels, the efficiency of, and the level of attendance at, outreach services were reported to benefit from the support of CHWs. As well as forgetting the second vaccine dose at 15 months, experienced mothers associated the dispensing of mosquito nets at 9 months (at the time of the first dose) with the end of childhood immunization, even after the second dose had been introduced. The main factor mentioned by Expanded Programme on Immunization and peri-urban health centre staff as having a negative effect on convenience was a long waiting time. In rural settings, time-saving strategies such as holding vaccination sessions on market days were regarded as having a positive impact on convenience and also on the provision of nutritional and family planning services. Finally, interviewees from all levels and settings said that paper-based data management made it difficult to monitor late immunizations and drop-outs (i.e. vaccine defaulters).

#### Caregivers’ perspective

Factors associated with vaccine hesitancy reported in interviews with caregivers in Ramba, Sovu and Gahanga were also categorized using the 3Cs framework (Table 3). With regard to confidence, caregivers reported no concerns about vaccine quality and trusted vaccines. Additionally, in rural settings, trust was reported to stem from respect for providers, including nurses and CHWs. CHWs were more often reported as a source of information in rural than in peri-urban settings. The impact of government information campaigns seemed greater in peri-urban settings.

**Table 3:** Caregivers’ comments on factors affecting vaccine hesitancy, Rwanda, 2018–2020

Factor affecting vaccine hesitancy^a^	Selected comments on factor by caregivers^b,c,d,e^	
	Rural health centres in Ramba and Suvo^f^ (*n* = 74)	Peri-urban health centre in Gahanga^g^ (*n* = 87)
**Confidence**		
Trust in the effectiveness and safety of vaccines and in their manufacturers	Positive: (i) all respondents were happy to vaccinate their child as it protects the child against disease (of course, selection bias must be considered here as caregivers at vaccination sessions were interviewed. However, respondents mentioned that, “nothing could stop me to come to the service” or called the services “a blessing,” which showed strong motivation and ample confidence); and (ii) hesitancy about vaccines from specific manufacturers was not observed or suspected as no respondent mentioned the name of a vaccine – they referred to the vaccine according to the time at which it needed to be administered (e.g. “vaccine for 2.5 months”)	Positive: hesitancy about vaccines from specific manufacturers was not observed or suspected as no respondent mentioned the name of a vaccine – they referred to the vaccine according to the time at which it needed to be administered (e.g. “vaccine for 2.5 months”)
Trust in, and personal experience of, the health system and health professionals	Positive: (i) health centre staff (mostly nurses and vaccinators) are seen as a good source of information (40 respondents) and are contacted to discuss questions on vaccination (29 respondents); and (ii) respondents mentioned that getting information on other health topics (e.g. stunting) during the vaccination sessions and receiving *shisha kibondo* (i.e. porridge) were additional benefits	Positive: health centre staff (mostly nurses and vaccinators) most frequently mentioned as a source of information (69 respondents) and as a contact for discussing questions (55 respondents). Negative: some mothers had negative experiences when their child was not given the vaccine (e.g. they were sent home because they were late or because a multidose vial could not be opened) or they received a fine (e.g. for being late, not having their child vaccinated or not re-engaging with the vaccination system after a home delivery)
**Complacency**		
Communication and media environment	Neutral: (i) radio is still a source of information on vaccination services (31 respondents); (ii) government campaign materials (e.g. flyers) were mentioned less often as a source of information (2 respondents); and (iii) interestingly, the vaccination card was also explicitly mentioned as a source of information (10 respondents)	Neutral: (i) radio is still a source of information on vaccination services (35 respondents); and (ii) government campaign materials (e.g. flyers) were frequently mentioned as a source of information (20 respondents), as were vaccination cards (6 respondents)
Influential leaders, immunization programme gatekeepers and vaccination lobbies	Positive: no caregivers mentioned local leaders as a negative influence (one respondent reported how the local leader goes around the village with a loudspeaker to give information about vaccination and other activities)	None
Historical influences	Positive: measles outbreak in 2019 increased the number of visible cases	None
Religion, culture, gender and socioeconomic factors	Positive: respondents mentioned that, when they are sick, there are people in the community (e.g. husband, CHW or neighbour) who can take the child for vaccination (i.e. community engagement). Negative: (i) caregivers mentioned that a few caregivers do not bring their children to the vaccination session, describing them as “careless” (3 respondents) or “busy with life” (1 respondent); and (ii) family conflict (1 respondent) and children who cry during the night after vaccination (1 respondent) were mentioned as reasons for not attending vaccination	Positive: some respondents mentioned that being sick was a barrier (4 respondents) but others (4 respondents) said that, even when they are sick, there are people in the community (e.g. husband, CHW or neighbour) who can take the child for vaccination (i.e. community engagement). Negative: some caregivers said that others missed appointments due to “carelessness” (11 respondents), work (2 respondents) or giving birth at home (6 respondents) but they often believed they would come later (e.g. after a visit from a CHW)
Knowledge and awareness	Positive: (i) CHWs were mentioned most frequently as a source of information on vaccination services (67 respondents) and as a contact for asking questions about services (70 respondents) but they were also thought important for community mobilization and follow-up (4 respondents); (ii) CHW follow-up was explicitly mentioned as having improved over the years (3 respondents) – “In the past, you could even not finish all vaccines/appointments and there was no one to follow-up on you but if you do not come as per your appointment in these days, a CHW will reach out to you and ask why you did not go for vaccination and advise you on how to catch up”; and (iii) other community members (mostly other mothers and neighbours) were frequently mentioned as sources of information on vaccination services (16 respondents)	Positive: (i) respondents mentioned that community members were aware of the importance of vaccination because of community mobilization, involving, for example, CHWs and campaigns; and (ii) CHWs were mentioned as the second most frequent source of information on vaccination services (45 respondents) and as a contact for asking questions about these services (44 respondents)
Perceived risks and benefits	Positive: (i) the need for disease prevention was strengthened by knowledge of cases of illness or death due to vaccine-preventable diseases (25 respondents); and (ii) all respondents were highly motivated to attend vaccination sessions by their desire to prevent disease or ensure their children will grow up to be healthy	Positive: in addition to their role in preventing disease, vaccines were trusted because they had had no negative effects so far (7 respondents). Negative: (i) fear of adverse effects following immunization (2 respondents); and (ii) most respondents did not know of cases of illness or death due to vaccine-preventable diseases – known cases (7 respondents) were mostly polio or measles, often in older people
Immunization as a social norm	Positive: respondents who did not know of any cases of illness or death due to vaccine-preventable diseases (38 respondents) mentioned that the community and their parents knew the importance of vaccines for protecting children; (ii) vaccination seemed to be standard (e.g. “every kid is vaccinated in the community”); and (iii) vaccination was frequently endorsed by community members, such as friends and family	None
**Convenience**		
Availability of the immunization service (including vaccine availability)	Positive: (i) only one mother mentioned the availability of vaccines as an explicit reason for coming to the vaccination session; and (ii) stock-outs were not mentioned as a barrier. Negative: not all vaccines were available at outreach posts (one respondent said that the vaccine given when the child is 15 months of age was not available)	Negative: caregivers can be sent away if they do not have an appointment, are late or if the desired vaccine or antigen is not offered that day, for example, to save multidose vials
Affordability of the immunization service	Negative: (i) more financial support requested (4 respondents); and (ii) reward for being fully vaccinated requested (1 respondent)	Negative: cost of transport is a barrier (3 respondents)
Geographical accessibility	Negative: (i) road to the health centre is bad and heavy rainfall makes it difficult and risky to access the vaccination service (12 respondents); (ii) distance was mentioned as a barrier or as a problem that must be tackled (6 respondents), although the new health centre at Sovu and outreach improved this for some mothers (5 respondents); (iii) one mother said that outreach posts should be kept open because they were closed for a while; and (iv) one mother mentioned that vaccination services were close, thanks to outreach, but they still needed to walk for more than an hour to all other services (e.g. to give birth)	Positive: vaccination sessions were easy to reach and travel times were short but some mothers still requested additional outreach (5 respondents). Negative: heavy rainfall makes it difficult to attend (15 respondents)
Ability to understand (i.e. language and health literacy)	Positive: one mother mentioned she cannot read or write and asks family members or neighbours to read the data on the vaccination card.	Negative: fear of being fined for a non-institutional birth despite the fine no longer existing (1 respondent)
Quality of the service (perceived or real)	Positive: the majority of caregivers were satisfied with the current service and did not suggest changes or additions (41 respondents). Neutral: suggestions for improvements related to other services, such as providing vitamins (1 respondent) and providing *shisha kibondo* (porridge) to all children and not only to some, as this is perceived as unfair (4 respondents). One respondent said: “Due to family planning, recent development and sensitization, those who did not respect family planning and kept giving birth or delivering are always receiving fortified foods or food supplements while, for us who respected it, are somehow punished and do not get access to food supplements and yet we still have children. Our wish and request is to change this decision and give fortified foods to all of us instead of punishing us”	None
Convenient time (including waiting time), place and cultural context	Positive: a desire to respect the given appointment is the second most frequent reason for attending a specific session (41 respondents) after the desire to “protect children against diseases,” which suggests that the appointment system, aided by vaccination cards, is effective. Negative: (i) clean water, electricity or both requested at the health centre (2 respondents); (ii) long waiting times (2 respondents); and (iii) the need to reduce waiting times compared with previous sessions (2 respondents)	Negative: (i) long waiting time due to too few nurses was a big issue (42 respondents); (ii) as a result, the waiting room was crowded and people had to wait outside; and (iii) one respondent suggested that the morning hours are better as it is less hot to walk with a child to the health centre
Design of vaccination programme and vaccination schedule	Neutral: mothers with more than one child mentioned that the vaccination schedule and vaccines had changed since they had their first child (e.g. an extra vaccine is needed when the child is 15 months old and two or three injections are required instead of one or two)	None

CHW: community health worker.

^a^ Factors were categorized using the 3Cs framework as related to confidence, complacency or convenience.^8^

^b^ Comments were classified as positive, neutral or negative with regard to their implications for vaccine uptake.

^c^ The numbers in the table refer to the number of interviewees who agreed with the comment.

^d^ Children’s caregivers were interviewed during vaccination sessions at health centres and outreach posts in two communities.

^e^ Details of which interviewees made each comment are available from the data repository.^32^

^f^ The Ramba health centre is in Ngororero District. After a measles outbreak in 2019, one of the health centre’s outreach posts became the Sovu health centre.

^g^ The Gahanga health centre is in the Kicukiro District of the Municipality of Kigali.

With regard to complacency, caregivers did not mention misinformation on social media in either setting. Carelessness and forgetting were considered to be the main reasons for underimmunization, particularly when mothers had older children, more tasks and different priorities.

Factors affecting convenience varied considerably between rural and peri-urban settings, with differences in travel distances and waiting times. In the rainy season, travelling safely with young children was complicated in rural areas and immunization was delayed; some people relied completely on outreach services. 

#### Comparing perspectives

Both service providers and caregivers largely agreed on the strengths of the vaccination programme but identified different challenges (Box 1, based on Table 2 and Table 3). By considering caregivers’ insights, immunization providers were able to obtain a broader perspective on their services, which in turn provided a basis for designing future interventions. For example, the introduction of an efficient digital data management system could help tackle the multiple challenges perceived by immunization providers while maintaining an appointment system well regarded by caregivers.

Box 1 **Vaccination service providers’ and caregivers’ perspectives on the strengths of, and challenges to, the measles vaccination programme in rural and peri-urban Rwanda, 2018–2020**

**Strengths**

All interviewees:

- CHWs provide information, mobilize caregivers and follow up vaccines;
- health centre staff provide information;
- confidence in vaccination is high;
- there is an intrinsic motivation to improve health; and
- community support.

Vaccination service providers:

- health ministry committed to supporting CHWs and mother-and-child health weeks.

Caregivers:

- vaccination cards;
- information provided by radio;
- high service quality; and
- appointments system.

**Challenges**

All interviewees:

- outreach services needed for poor and hard-to-reach families.

Vaccination service providers:

- high staff turnover and workload and need for training;
- tracing of vaccine defaulters inefficient;
- outreach services for measles and rubella vaccination inadequate; and
- mothers missing the second measles and rubella vaccine dose for their children.

Caregivers:

- difficult vaccination access in the rainy season;
- long distances to health centres; and
- long waiting times.

CHW: community health worker.

### Causal loop diagrams

**Fig. 2:**
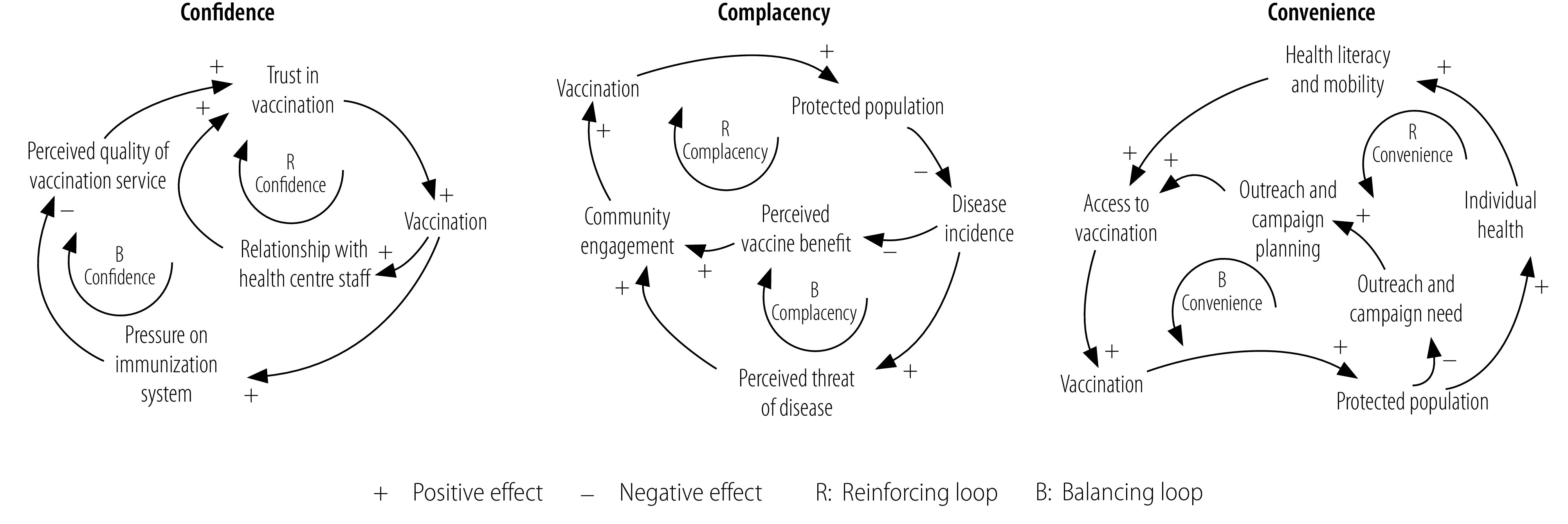
Causal loop diagrams of factors affecting vaccine hesitancy, by factor category, Rwanda, 2018–2020

Notes: Factors affecting vaccine hesitancy were categorized using the 3Cs framework as related to confidence, complacency or convenience.^8^ The R loops are reinforcing feedback loops that cause change in the same direction and the B loops are balancing loops that cause change in the opposite direction.

**Fig. 3:**
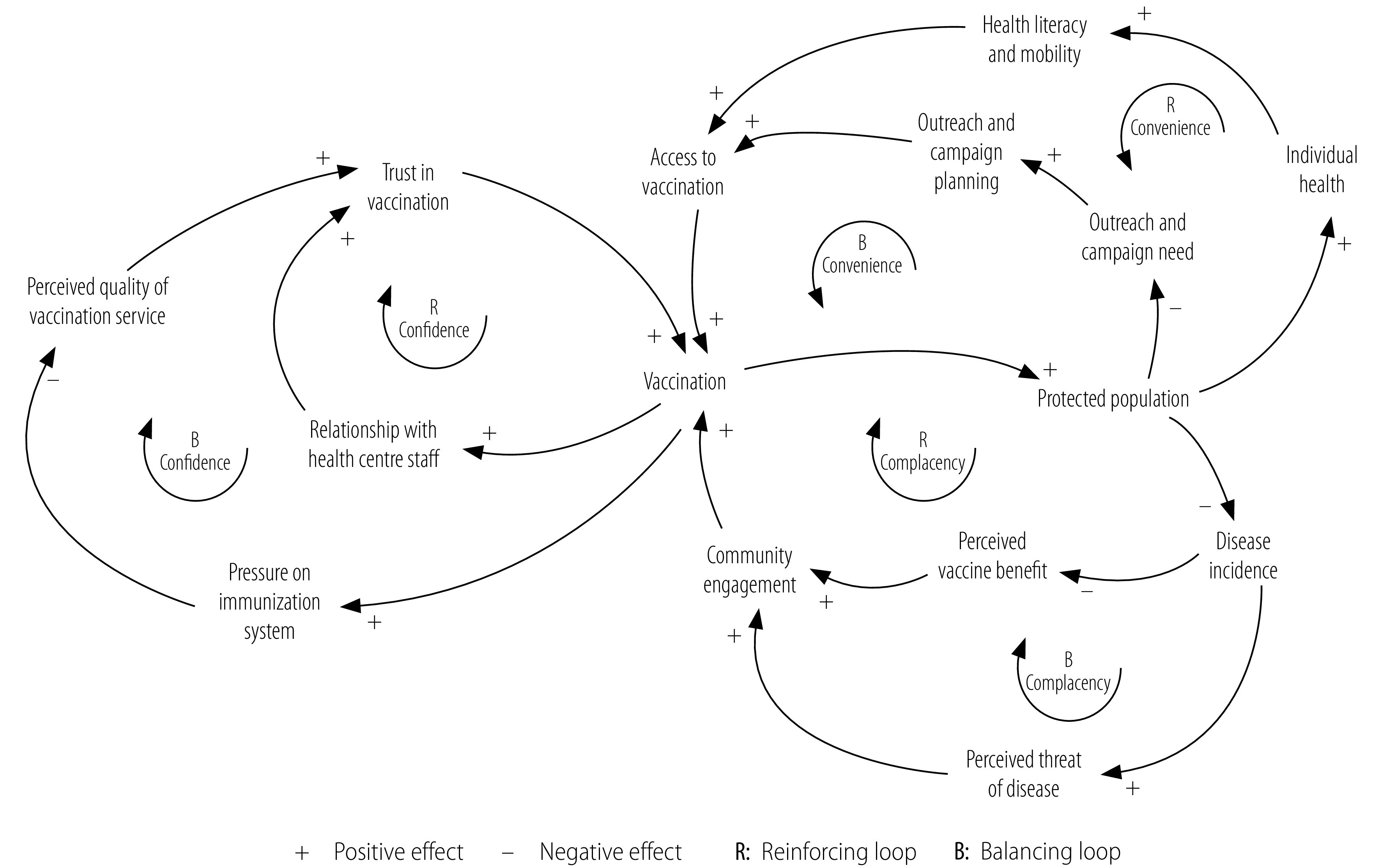
Composite causal loop diagram of factors affecting vaccine hesitancy, Rwanda, 2018–2020

Notes: This diagram is the composite of the three causal loop diagrams in Fig. 2. Factors affecting vaccine hesitancy were categorized using the 3Cs framework as related to confidence, complacency or convenience.^8^ The R loops are reinforcing feedback loops that cause change in the same direction and the B loops are balancing loops that cause change in the opposite direction.

The causal relationships between the main factors affecting vaccine hesitancy identified in interviews with vaccination service providers and caregivers are illustrated in three causal loop diagrams in Fig. 2 (available at https://www.who.int/publications/journals/bulletin/), for confidence, complacency and convenience, respectively. Fig. 3, which is a composite of these three diagrams, indicates that vaccine uptake is governed by three key factors: (i) trust in vaccination; (ii) community engagement; and (iii) access to vaccination. As vaccine uptake evolves, six feedback loops are activated (Fig. 3): three balancing B loops and three reinforcing R loops that can increase or decrease trust, engagement or access. These loops illustrate the dynamic nature of the system. The same factors and loops were identified in both rural and peri-urban settings but their relative impact on vaccination uptake differed. For example, in rural settings, access to vaccinations was reduced by a lack of outreach services and information campaigns, whereas, in peri-urban settings, workload pressure on the immunization system had a negative impact on trust in vaccination.

For confidence, reinforcing factors represented by the R loop include: (i) respectful relationships with health centre staff and community health workers engenders trust in vaccine services; (ii) mothers overcome fear of being late for immunization when they know the nurse and feel welcome; (iii) multiple contacts between the caregiver and nurse increases trust; and (iv) an entry in the mother-and-child booklet indicating immunization has been completed is encouraging for both caregivers and their families. Balancing factors represented by the B loop include: (i) long waiting times; (ii) short contact times between nurse and caregiver, which give the nurse little time to explain vaccination (one reason was numerous administrative tasks and demands from other vaccination programmes); and (iii) suspension of educational sessions before vaccinations during the coronavirus disease 2019 pandemic.

For complacency, reinforcing factors represented by the R loop include: (i) a well-protected population experiences fewer cases of disease, which increases the perceived benefit of vaccination and community engagement. Balancing factors represented by the B loop include: (i) a decline in cases of disease lowers both awareness of the threat to young children and the perceived importance of vaccination; and (ii) caregivers worry less about their children’s health as they grow up, which increases complacency and can lead some to deprioritize their vaccination appointments.

For convenience: reinforcing factors represented by the R loop include: (i) families that are healthy, literate and financially stable are more likely to understand their appointment schedule and can afford to travel to health centres to vaccinate their children. Balancing factors represented by the B loop include: (i) the risk of a disease outbreak is increased in poorly protected populations (which can be remedied by immunization campaigns and enhanced vaccination services, including outreach).

To maintain measles vaccination coverage at the desired level of over 95%, behaviour that increases vaccine uptake, as indicated by loops in the causal loop diagram (Fig. 3), must be actively promoted by health interventions. For example, government-led information and vaccination campaigns, community advocacy and direct communication from CHWs during home visits all regularly boost the perceived benefit of vaccination and community engagement. The disruption caused by the coronavirus disease 2019 (COVID-19) pandemic affected the 3Cs: (i) convenience was diminished because vaccination sessions were smaller and less accessible; (ii) confidence was reduced by heightened pressure on the system and fear of infection at the health centre; and (iii) complacency was increased by less contact between CHWs and caregivers.

**Fig. 4:**
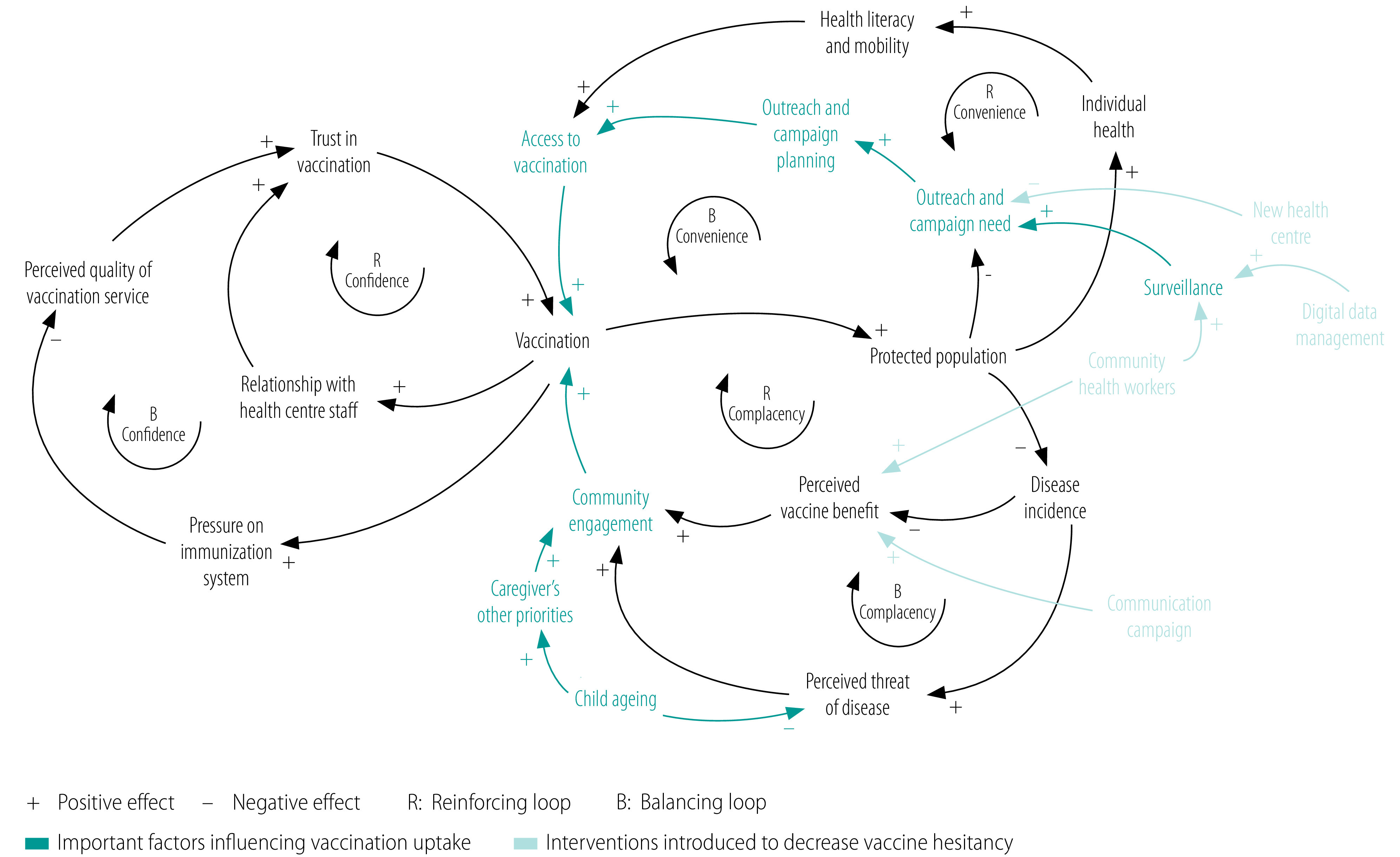
Composite causal loop diagram of factors affecting vaccine hesitancy during a measles outbreak, Rwanda, 2019

Notes: The measles outbreak occurred in the catchment area of the Ramba health centre in 2019. This diagram is an adaptation of the causal loop diagram in Fig. 3. Factors affecting vaccine hesitancy were categorized using the 3Cs framework as related to confidence, complacency or convenience.^8^ The R loops are reinforcing feedback loops that cause change in the same direction and the B loops are balancing loops that cause change in the opposite direction.

The composite causal loop diagram can also be used to illustrate the circumstances that led to the 2019 measles outbreak in Sovu (Fig. 4). First, community and caregiver engagement with the second measles and rubella immunization dose for children aged 15 months was low because mothers had other priorities, perceived their children as stronger due to being older, or were not aware of the second dose. Second, in the absence of outreach services, health centres did not know which children were underimmunized. Therefore, the need for an additional immunization campaign or outreach services was not recognized. The resulting outbreak triggered a new immunization campaign and further investigations. Eventually, a new health centre was established in the affected area, which reduced the previously underimmunized population’s dependency on both outreach services and immunization campaigns. The ongoing development of digital data management systems at health centres will support this change. In addition, CHWs can help bridge the gap between caregivers and the health system and can assist in surveillance.

## Discussion

Our analysis of factors influencing vaccine hesitancy in Rwanda offers several insights. For example, trust in vaccination and social cohesion are factors that can be leveraged in various settings. However, the differences we identified between rural and peri-urban settings in the ease of travel indicate that solutions for vaccine hesitancy and vaccine policy design are dependent on the setting, even for the same vaccine in the same country. Both vaccination service providers (i.e. Expanded Programme on Immunization staff and CHWs) and caregivers thought that the accessibility of vaccination sessions and the quality of the immunization service were important influences behind underimmunization. However, the insights we obtained from service providers and caregivers revealed they had differing perspectives, which provided an opportunity for collective learning and for increasing vaccine uptake.

Some service delivery policies had unintended consequences. For instance, according to national guidelines, multidose vaccine vials must be used at fixed sites rather than at both outreach posts and fixed sites to reduce wastage. This policy may increase vaccine hesitancy by reducing access to, or the convenience of, vaccination for people dependent on outreach services. Moreover, in some places in Rwanda, there used to be a financial penalty if a child was not registered with the vaccine system at birth. Consequently, families whose children were born at home were often deterred from visiting health centres due to misinformation that the penalty still existed. Conversely, this lasting fear of a financial penalty may incentivize some pregnant mothers to engage with vaccination.

Our findings illustrate the crucial role of community engagement in building system resilience. Causal loops can be strengthened or weakened by external elements, such as interventions made in response to a measles outbreak. For instance, communication within communities can be leveraged by prolonging CHW programmes, thereby enhancing social cohesion. In contrast, the COVID-19 pandemic induced the perception that health centre visits were unsafe. This perception, combined with the temporary cancellation of community awareness activities and educational sessions at health centres, resulted in immunization being delayed until mid-August 2020. The speed at which vaccine uptake was restored after the disruption showed the resilience of the system.^35^^,^^36^

As outreach services are a cornerstone of measles immunization programmes in some populations, their sustainability will affect future immunization programmes. The efficiency of outreach could be increased by focusing efforts on hard-to-reach populations or by establishing temporary outreach posts during the rainy season. Dependency on outreach services could be reduced by opening new health-care delivery points or by providing physical or financial support to enable caregivers to travel to health centres, both of which would enhance the sustainability of the immunization system.

Our study of two settings in Rwanda revealed leverage points that cut across several factors influencing vaccine hesitancy (i.e. one specific intervention can impact multiple loops within the immunization system). For example, we found that the connecting role of CHWs was pivotal and that they could function as a high-potential leverage point because they have a direct impact on two feedback loops in Fig. 3: the balancing convenience loop (i.e. identifying the need for outreach and vaccination campaigns) and the reinforcing complacency loop (i.e. increasing awareness of the benefits of vaccination through home visits).^25^ Furthermore, the digitization of local immunization data, such as the immunization status of the population (irrespective of the point of vaccination), could guide targeted and timely preventive interventions (e.g. catch-up vaccinations in the child’s second year of life), assist in campaign planning and help trace defaulters. Nevertheless, the factors affecting vaccine hesitancy can change over time (e.g. in response to vaccine-hesitant social media influencers).^37^

Our study has limitations. First, the presence of a well-functioning vaccination delivery system in Rwanda may make it more difficult to generalize our findings. Second, although we carefully selected the study settings, they were limited in number and our findings relate to only rural and peri-urban communities. More research is needed on urban and other settings. Third, we were not able to interview caregivers who are not present at vaccination sessions. However, interviewees were asked about the motivations of caregivers who missed sessions. Similarly, we interviewed only CHWs and other vaccination service providers who spoke on behalf of their teams, which may have introduced bias. Finally, the COVID-19 pandemic complicated fieldwork.

In addition to overcoming these limitations, future research could build on our insights and causal loop diagrams to develop a human-centred, collaborative approach to identifying leverage points that could be used to design sustainable interventions for minimizing vaccine hesitancy. Moreover, our systems thinking approach and factors influencing hesitancy could be integrated with initiatives like the Vaccine Confidence Project,^38^ which contains a tool for mapping confidence globally.^12^^,^^14^^,^^39^^,^^40^
